# COVID-19 in a Three-Year-Old Girl With Total Anomalous Pulmonary Venous Return: A Case Report

**DOI:** 10.7759/cureus.11768

**Published:** 2020-11-29

**Authors:** Kholod A Alfareh, Adnan Zafar

**Affiliations:** 1 Pediatrics, King Khalid Medical City, Abha, SAU; 2 Pediatrics Pulmonology, King Fahad Medical City, Riyadh, SAU

**Keywords:** total anomalous pulmonary venous return, tapvr, covid-19, sars-cov-2, congenital heart disease

## Abstract

Total anomalous pulmonary venous return (TAPVR) is a rare congenital heart disease (CHD) with an incidence of less than 1%. It is known that coronavirus disease 2019 (COVID-19) has a worse prognosis in those with underlying disorders. Children with congenital heart defects can contract COVID-19 irrespective of their surgical correction status. We report a case of a three-year-old girl with unoperated TAPVR, who presented with respiratory distress, lethargy, and reduced feeding. Reverse transcription polymerase chain reaction (RT-PCR) of nasopharyngeal aspirate came back positive for severe acute respiratory syndrome coronavirus 2 (SARS-CoV-2). There was no growth of any other viral or bacterial pathogens. Throughout her admission, she had an overall mild course of the disease and did not need mechanical ventilation. Oxygen was given via nasal cannula to maintain SpO_2_ in the target range. Chest X-ray (CXR) showed bilateral patchy consolidation while a chest CT with contrast showed significant venous congestion. Her length of hospital stay was 25 days. Infection with SARS-CoV-2 did not cause a critical disease and was not different clinically to any other bacterial or viral infection. The potential risk of further cardiac deterioration in COVID-19 in any CHD should be handled with caution as these children can decompensate rapidly.

## Introduction

Coronavirus disease 2019 (COVID-19) poses clinical and social challenges for managing children with underlying conditions including congenital heart disease (CHD). Preventive measures in the wake of infection have resulted in a reported decrease in ED visits by up to 50% in some institutes [1]. Many CHD correction surgeries have been delayed during the beginning of the pandemic as medical personnel were still in the midst of exploring the virus [1-2]. Regardless, healthcare workers are in need to reach this group of individuals in order to make sure follow up is properly done [1]. Total anomalous pulmonary venous return (TAPVR) is characterized by having all four pulmonary veins drain into the systemic venous circulation [2]. It is often accompanied by other congenital anomalies, such as patent foramen ovale (PFO) [3]. Even though surgical interventions could decrease the overall rate of mortality in this group, the patients are still prone to severe pneumonia and death [4]. In this case we present a child with uncorrected TAPVR who contracted COVID-19.

## Case presentation

**Figure 1:**
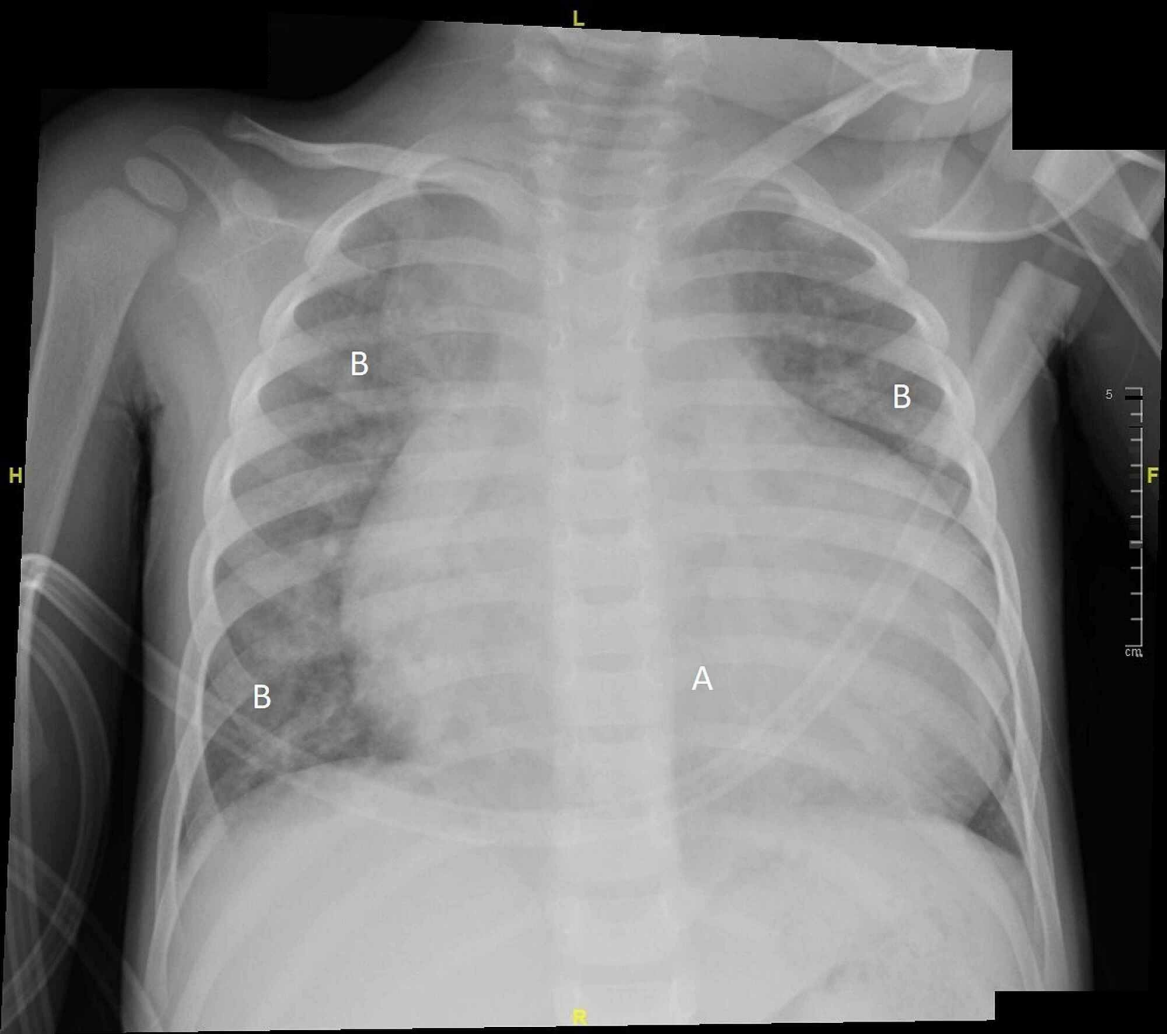
Initial CXR in the ED showing consolidation and cardiomegaly. A: cardiomegaly; B: lung consolidation CXR, chest X-ray

We report the first case of a three-year-old-girl with uncorrected TAPVR, patent ductus arteriosus (PDA), and an unrestricted PFO with positive COVID-19. She was diagnosed with TAPVR soon after birth and the doctors advised the parents for surgical correction, but they refused to proceed with surgery. She was on long-term therapy by furosemide (Lasix) due to a dilated right ventricle with suprasystemic pressure. The patient presented to our hospital after being transferred from a district general hospital to the ED of our tertiary care hospital due to suspicion of COVID-19. On presentation, her mother provided a history of cyanosis, dyspnea, and decreased activity in the preceding days with no history of fever, cough, sneezing, or runny nose. Screening for SARS-CoV-2 in the ED by reverse transcription polymerase chain reaction (RT-PCR) of the nasopharyngeal secretions came back positive. Surprisingly, contact tracing revealed no direct contact with any positive source. Afterwards, the patient was isolated and complete thorough examination was carried. Regarding physical examination, the patient was slightly pale, hemodynamically stable, and her SpO2 80%. Her chest examination indicated there was bilateral wheezing without crepitations and her accepted oxygen saturation (SpO2) as advised previously by the cardiology team was ≥75%. Furthermore, cardiac examination was performed by the pediatric cardiology team and was noted to be of her usual state with no new abnormality. Initial chest X-ray (CXR) showed bilateral patchy consolidations and cardiomegaly with no signs of pleural effusion (Figure 1).

**Figure 2:**
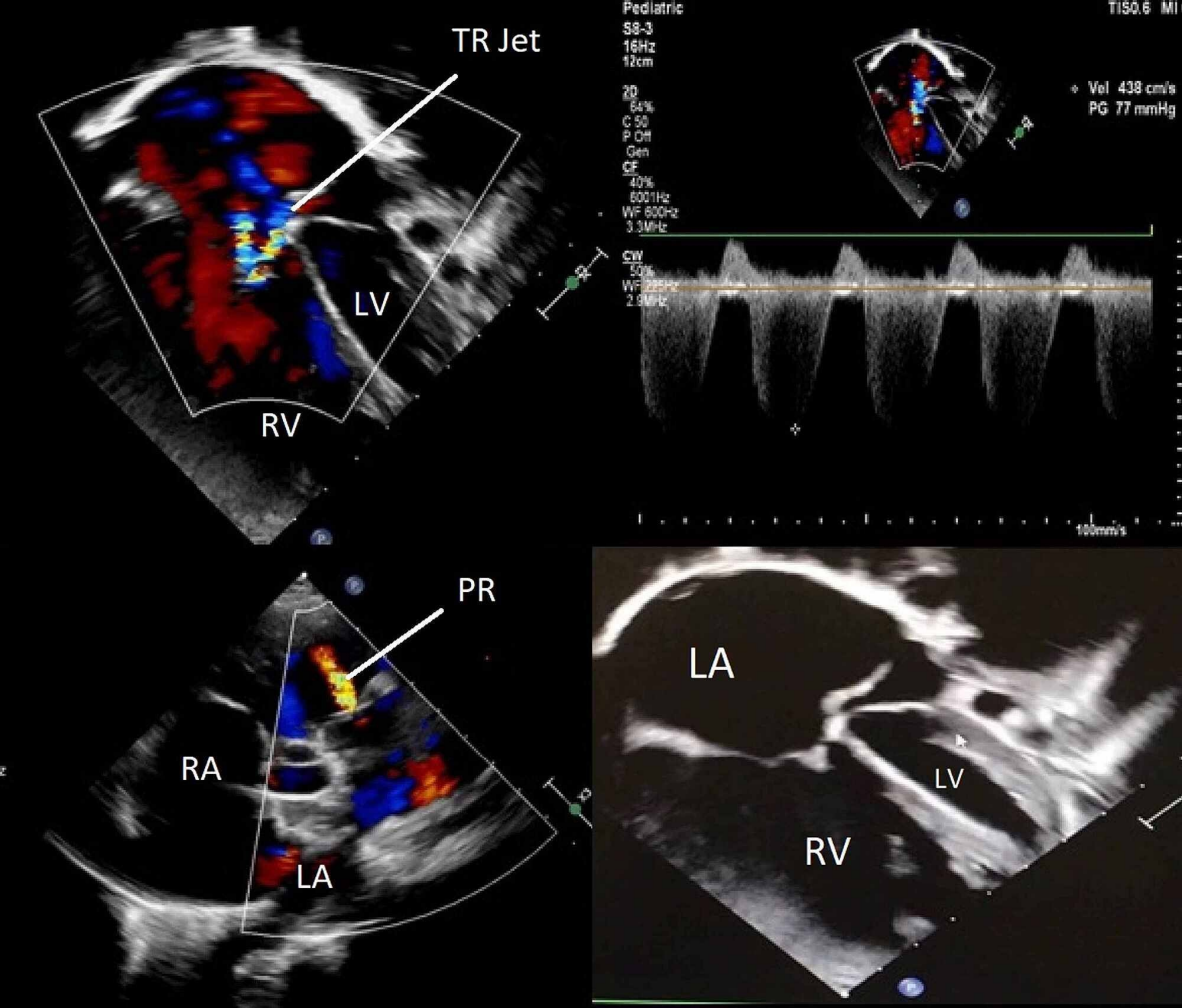
Echocardiography showing dilated IVC with flow reversal, dilated RA, moderate TR with maximum gradient of 77 mmHg. AV: aortic valve; LA: left atrium; LV: left ventricle; PR: pulmonary regurgitation; RA: right atrium; RV: right ventricle; TR: tricuspid regurgitation; TV: tricuspid valve; IVC: inferior vena cava

On further investigation, an electrocardiogram (ECG) demonstrated sinus tachycardia with no evidence of ventricular hypertrophy or T wave changes. Arterial blood gas (ABG) was consistent with respiratory acidosis. Afterwards, an echocardiography was performed which showed dilated inferior vena cava (IVC) with flow reversal, dilated right atrium, stretched PFO (right to left shunt), and moderate tricuspid regurgitation with maximum gradient of 77 mmHg. There was moderate pulmonic insufficiency, dilated main pulmonary artery, grossly dilated right ventricle, and squashed left ventricle (Figure 2).

She was admitted to the cardiology ward for observation and supportive care. All cultures, such as blood, pharyngeal and urine were negative for any growth. Inflammatory markers including C-reactive protein (CRP) and erythrocyte sedimentation rate (ESR) were within normal range. During her hospital stay of 25 days, she was stable with low dependency care and no clinical deterioration was observed. Initially, due to her susceptibility to infections, empirical prophylactic antibiotic was given (Ceftriaxone) which was discontinued after five days once the blood culture reported no growth. For the wheezy chest, a salbutamol inhaler was given every six hours and demonstrated a good response. She needed 1.5-2 L/min oxygen by nasal cannula to keep oxygen saturations in the target range (Table 1).

**Table 1:** Respiratory status and SpO2 assessment throughout admission. ^a^Oxygen requirement by face mask. ^b^Oxygen requirement by nasal cannula SpO_2_: oxygen saturation

Day	Day 1 (ER)	Day 4	Day 14	Day 16	Day 20	Day 25 (discharge)
Oxygen	5 L^a^	1.5 L^b^	1.5 L^b^	1.5 L^b^	2 L^b^	0.5 L^b^
SpO_2_	92%	92%	84%	75%	82%	88%
Heart rate	140	120	120	105	100	95

**Figure 3:**
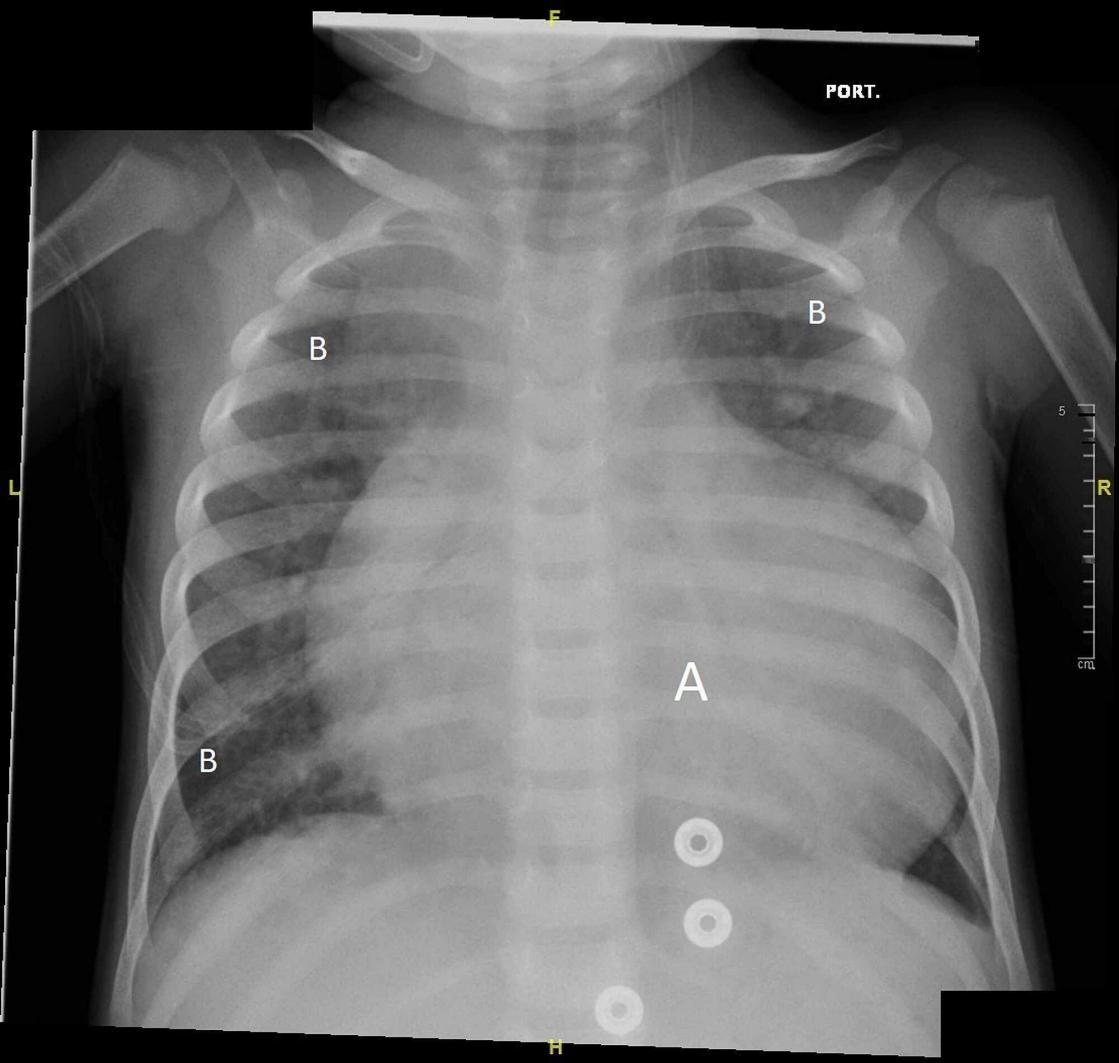
Second CXR on the fifth day of admission. A: cardiomegaly; B: improvement of chest consolidation CXR, chest X-ray

All initial blood work was normal except troponin I, brain natriuretic peptide (BNP), and lactate dehydrogenase (LDH) (Table 2). Troponin I and BNP levels decreased over the course of her illness. A second CXR was done on the fifth day, which showed gross cardiomegaly, bilateral lung congestion, and bilateral lower lobar atelectasis findings (Figure 3). This did not impact the clinical status and no escalation in respiratory support was needed. A second nasopharyngeal aspirate (NPA) on the fifth day remained positive while a third NPA on day 11 was reported negative for SARS-CoV-2.

**Table 2:** Troponin, BNP, LDH, and COVID-19 PCR throughout the admission. BNP: brain natriuretic peptide; LDH: lactic acid dehydrogenase;  RT-PCR: reverse transcription polymerase chain reaction; SARS-CoV-2: severe acute respiratory syndrome coronavirus 2

Day	Day 1 (ER)	Day 5	Day 11
Troponin	39.3 ng/L	42.8 ng/L	20 ng/L
BNP	6824 pg/mL	6436 pg/mL	4926 pg/mL
LDH	720 IU/L	568 IU/L	240 IU/L
RT-PCR (SARS-CoV-2)	Positive	Positive	Negative

**Figure 4:**
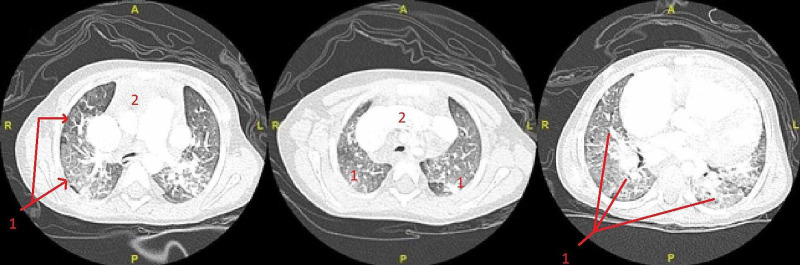
CT of the chest with IV contrast utilizing the cardiac great vessels protocol. 1: chest consolidation; 2: cardiomegaly

Prior to discharge, a CT scan of the chest was done with IV contrast to assess the great cardiac vessels which revealed gross cardiomegaly, significantly prominent superior vena cava, prominent left brachial cephalic vein, an abnormal vertical venous channel connecting the left brachial cephalic with the left atrium, anterior septal defect (ASD), and extensive pulmonary venous congestion (Figure 4).The patient was discharged on home oxygen and an angiotensin-converting enzyme inhibitor (Captopril) was started by the cardiology team. A close follow-up appointment was given to wean oxygen, and after further effort to convince the parents to accept surgical correction, the patient’s family consented for it.

## Discussion

In April 2020, the American Heart Association and the American Academy of Pediatrics published a joint guidelines to help deal with cardiac complications of COVID-19 [5]. Generally, adult patients with underlying cardiac disease have been found to have a worse outcome after contracting COVID-19 compared to healthy individuals [6]. However, some pediatric studies reported a milder course of disease in CHDs [7, 8]. Regardless, caring for pediatric patients with CHD should be approached with a well-constructed plan during the pandemic. Dong et al. devised a streamlined diagnostic pathway for children with cardiac involvement in the presence of CHD; this involves CXR, CT chest, cardiac enzymes, and echocardiogram [9]. The European Association of Cardio-Vascular Imaging recommended that cardiac imaging when needed in COVID-19 patients should ideally be done at the patient’s bedside with minimal exposure to reduce the risk of infection [10]. It has been found that 80% of TAPVR patients without corrective surgery die due to progressive congestive heart failure, whereas patients with corrective surgery have a longer life expectancy [3, 11].

For our patient, troponin I and BNP levels were elevated in the initial phase after admission indicating likely myocardial involvement. In pediatrics, there is limited data with regard to interpretation of troponin levels for those who are infected with SARS-CoV-2. However, some case reports have reported its utility as a disease marker for children with COVID-19 [12-13]. Multiple theories have been put forward to describe the mechanism of myocardial injury. They postulate either a direct role of the virus as seen in previous SARS infections or hypoxia-induced injury and an increased inflammatory cascade [14-16]. A CT of the chest in children with COVID-19 has been mostly done when there is disease progression or where CXR does not correlate well with the clinical picture. It has been discouraged as a screening tool [17]. It was done for our patient to plan corrective surgery in the future. It is important to maintain oxygen saturations in a predetermined range in CHD patients as over or under oxygenation can have deleterious effect [18]. We started oxygen at admission and later weaned it appropriately in accordance with the advice given by the cardiologist. All nasopharyngeal swabs were taken in line with the guideline published by the World Health Organization. The decision to discharge the patient was collectively made as a team once NPA was negative for SARS-CoV-2 and there were no clinical concerns.

To the best of our knowledge, this is the first case of uncorrected TAPVR with COVID-19 that had a mild course of the disease without any complication. Children with CHD remain susceptible to being infected with SARS-CoV-2 but mostly have a mild disease. Careful approach can prevent further cardiac deterioration and prevent secondary chest infections. Despite some good evidence, further studies are needed to fully understand impact of COVID-19 on CHD in the pediatric population [19].

## Conclusions

In conclusion, pediatric groups with CHD are challenging to treat while over the course of the pandemic it was shown that such a group usually have a mild course of disease when contracting COVID-19 but are more susceptible to being infected with it even when contact tracing is negative for COVID-19 contact. Lastly, careful observation and approach should be carried as a multidisciplinary team to prevent further cardiac deterioration and prevent secondary chest infections.
